# An overview of the epidemiology and emergence of influenza A infection in humans over time

**DOI:** 10.1186/s13690-017-0182-z

**Published:** 2017-03-27

**Authors:** Chau Minh Bui, Abrar Ahmad Chughtai, Dillon Charles Adam, C. Raina MacIntyre

**Affiliations:** 1 School of Public Health and Community Medicine, University of New South Wales, Sydney, NSW 2052 Australia; 2 College of Public Service & Community Solutions, Arizona State University, Phoenix, AZ USA

**Keywords:** Influenza A, Emerging infectious diseases, One health, Avian influenza

## Abstract

In recent years multiple novel influenza A strains have emerged in humans. We reviewed publically available data to summarise epidemiological characteristics of distinct avian influenza viruses known to cause human infection and describe changes over time. Most recently identified zoonotic strains have emerged in China (H7N9, H5N6, H10N8) – these strains have occurred mostly in association with visiting a live bird market. Most zoonotic AIVs and swine influenza variants typically cause mild infections in humans however severe illness and fatalities are associated with zoonotic H5N6, H10N8, H7N9 and H5N1 serotypes, and the H1N1 1918 Spanish Influenza. The changing landscape of avian influenza globally indicates a need to reassess the risk of a pandemic influenza outbreak of zoonotic origin.

**Electronic supplementary material:**

The online version of this article (doi:10.1186/s13690-017-0182-z) contains supplementary material, which is available to authorized users.

## Background

Following the emergence of the zoonotic influenza A H5N1 in humans in 1997, Hong Kong, there was global concern that the virus would evolve to become capable of human to human transmission causing a pandemic similar to the 1918 Spanish flu pandemic which killed over 50 million people [1]– this concern stemmed from the high case fatality rates (CFR) and absence of natural herd immunity to the H5 hemagglutinin (HA).

Most high-level international activity has been focused on global pandemic preparedness and how to manage emerging infectious diseases (EIDs) once they have occurred – in recent years there has been a shift in focus in pandemic planning to prevent zoonotic infections at the level of the species jump to eliminate the potential for zoonotic transmission before the pathogen can establish itself in humans. Jones et al. (2008) [2] provide key research on this subject. The authors compiled a comprehensive listing of all EID events in humans from 1940 to 2004 to identify drivers of infectious disease emergence. The study predicted that most zoonotic EID events are correlated with human population density and growth and latitude, rather than areas with abundant wildlife species. However, only *one* of the 335 identified global EID events was an influenza A virus (the H5N1 subtype). In recent years *multiple* novel reassortant influenza A viruses have emerged: H5N6 in 2014, H7N9, H10N8 and H6N1 in 2013, and a novel H1N2 swine-flu variant in 2012.

We previously compared the novel H7N9 which emerged in humans in Shanghai, 2013 to the more extensively studied H5N1 and highlighted some puzzling differences in the epidemiology [3]. In this brief report, we compared the epidemiology and emergence of *all* influenza A serotypes known to cause human infections. The aim of this report is to identify trends or changes in the epidemiology and characteristics of zoonotic influenza A emergence in humans over time.

## Methods

For all zoonotic avian influenza virus AIV serotypes except H5N1 and H7N9 (for which methods have been previously described ([3]), we reviewed epidemiological features of outbreaks or cases. We searched publications using the Scopus database, and grey literature using the World Health Organisations (WHO), Centres for Disease Control and Prevention (CDC), Flutrackers and ProMed websites, using the subtype as the keyword. Materials were limited to those which were published in the English language. Publications from Scopus were searched in February to March 2015 (and again in August to September 2016) for relevant studies. Publications were not limited by study design or year of publication. We retrieved all articles with the subtype (e.g. “H5N6” or “H9N2”) in the title and identified relevant articles through scanning titles, and then abstracts. Further relevant studies were identified by examining the reference lists of relevant articles.

We extracted information on year and country of incidence, characteristics of person/s affected (sex, age, occupation), clinical signs, mortalities, details of animal exposure and pathogenic classification of virus. Highly pathogenic (HPAI) outbreaks in poultry typically cause acute, severe mortalities and have significant economic implications due to the severe control measures (e.g. mass culling, disinfection, quarantine and movement restrictions) imposed following outbreaks. Low pathogenic (LPAI) outbreaks in poultry occur more commonly, have less clinical significance, and are harder to detect. Extracted information is provided in Additional file 1: Table S1, last updated September 2016.

Details of individual outbreaks and isolated cases were not extracted for non-zoonotic influenza A strains which have caused human infections (seasonal and pandemic influenzas), as it is outside the scope of this study to record details on the enormous amount of human influenza cases, and their epidemiology have been detailed extensively in previous review articles [1, 4]. Similarly, extensive reviews of swine influenza cases have already been published elsewhere [5, 6].

**Fig. 1:**
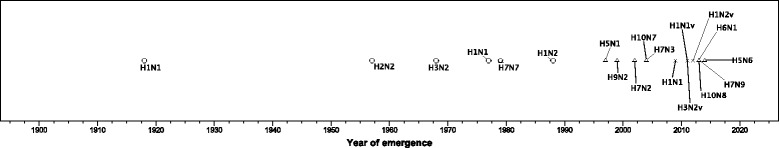
Timeline of Influenza A serotype emergence by year and zoonotic host from 1918 to 2015. Each point indicates a distinct avian influenza serotype known to have caused human infection, placement of point corresponds to their year of emergence. The different point shapes correspond to the animal host which was identified when the serotype was first reported in humans: a pentagon corresponds to a seal host, a triangle corresponds to avian host, a cross corresponds to swine host, and a circle corresponds to instances where the animal host has not yet been identified

We summarised features of all human and zoonotic serotypes of influenza A which have been documented to cause human infections (Table 1, last updated September 2016) and produced a timeline of the emergence of influenza A serotypes in humans starting from the 1918 Spanish flu pandemic (Fig. 1). The emergence of variant strains of H3N2, H1N2, and H1N1 were treated as separate emergent events. Figure 1 was created using IBM SPSS Statistics for Windows (v22.0).

**Table 1:** Epidemiological features of known avian influenza serotypes which have caused human infections

Avian influenza serotype	te (month, year) and location of first report in humans	Number of human cases	Number of deaths	istory of animal exposure in human cases	uration of persistence in human population	Extent of Human to human transmission	Classification of pathogenicity	Animal hosts/potential animal hosts
H5N6	Feb-2014 Changsha city, Hunan, China [1]	15	9 reported deaths	Animal exposure	Sporadic. Last case reported in 2016. [2]	None	HPAI	Avian [3] [4] Feline [5]
H10N8	Dec-2013 Nanchang, Jiangsu, China [2]	3 [2]	2 reported deaths [2]	Animal exposure [2]	Sporadic. Last case reported in 2015. [2]	None	LPAI	Avian [3] [4]
H6N1	May-2013 Taiwan [6]	2 [7]	No reported deaths	None [6]	Isolated event	None	LPAI	Avian [8] [4]
H7N9	Mar-2013 Minhang district, Shanghai, China [9]	791 [2]	314 reported deaths [10]	Animal exposure [11]	Seasonal occurrence. Last reported in 2016 [2]	Limited (41 reported occurrences) [12–14]	LPAI	Avian [4] Canine [15]
H1N2v	Jun-2012 Minnesota, United States [16]	8 [17]	No reported deaths	Animal exposure [16]	Sporadic. Last reported in 2016	None	n/a	Swine [16, 18]
H1N1v	Dec-2011 Winconsin, United States	20 [17]	No reported deaths	Animal exposure	Sporadic. Last case in 2015	None	n/a	Swine [18]
H3N2v	Indiana, United States Jul-2011	372 [17, 19]	1 death [19, 20]	Animal exposure [20]	Sporadic. Last case in 2016 [19]	Limited (15 possible occurrences) [20]	n/a	Swine [18, 21]
H10N7	Mar-2010 Australia [22]	2 [22]	No reported deaths	Animal exposure [22]	Isolated event	No	LPAI	Avian [4] Pinnipeds [23] Racoons [24]
H1N1pdm09	Mar-2009 Mexico, Canada, US [21]	Estimated > 24% population in 19 countries	Estimated 0.02% of cases result in death [25]	Not described [18]	2009 to current	Widespread	n/a	Swine [18] Pinnipeds [26]
H7N3	Mar-2004 British Columbia, Canada [27]	19	No reported deaths	Animal exposure	Sporadic cases. Last reported in 2012 [28]	None	LPAI/HPAI	Avian [29] [4]
H7N2	Oct-2003 Westchester county, United States [30]	16	No reported deaths	Animal exposure	Sporadic cases. Last reported in 2007 [31]	None	LPAI	Avian [4, 29] Swine [32]
H1N2	Oct-2000 Singapore [33, 34]	No global estimates	No deaths attributable to this subtype (UK) [33]	Not described	2000-2003 [33]	Widespread	n/a	None
H7N7	Dec-1979 Boston, United States [35]	94	1 reported death.	Animal exposure	Sporadic cases. Last reported in 2013 [36]	Limited (3 reported occurrences) [37]	LPAI/HPAI	Avian [4] Equine [38] Pinnipeds [35]
H9N2	Jun-1999 Hong Kong [39]	28	No reported deaths	Animal exposure	Sporadic cases. Last reported in 2016	None reported	LPAI	Avian [40] Swine [4, 41]
H5N1	Feb-1997 Hong Kong [9]	850 [2]	449 reported deaths [2]	Animal exposure [42]	Sporadic cases. Last reported in 2016. [2]	Limited (41 reported occurrences) [42]	HPAI	Avian Canine Feline Swine Stone marten [4, 9]
H1N2	Dec-1988 China [34, 43]	No case estimates (19 isolates were recovered from China) [34, 43]	No deaths attributable [43]	Not described [16, 43]	Sporadic cases from Dec-1988 to Mar-1989 [43]	Widespread	n/a	Swine [16]
H1N1 (seasonal flu)	May-1977 Tianjin, China [44]	Estimated 3–5 million cases (severe illness) per year [45]	Estimated 250 000 – 500 000 deaths per year [45]	Not described	Seasonally endemic (1977 to current) [46]	Widespread	n/a	None
H3N2 (Hong Kong flu)	Jul-1968 Hong Kong	No global estimates	No global estimates	Not described	Pandemic; seasonally endemic and sporadic (1968 to current)	Widespread	n/a	Swine [47] Canine [48]
H2N2 (Asia flu)	Feb-1957 Guizhou, China [49]	No global estimates	Estimated 1.1 million deaths [50]	Not described	Pandemic; seasonally endemic and sporadic (1957 to 1968) [51]	Widespread	n/a	None
H1N1 (Spanish Flu)	Aug-1918 North America, Europe & Africa [52]	Estimated 500 million cases [52]	Estimated 50 million deaths [52]	No described	Pandemic; seasonally endemic and sporadic (1918 to 1957) [52]	Widespread	n/a	None

Information from this table was obtained from Additional file 1: Table S1 unless indicated otherwise. Numbers of human cases and deaths are total global counts or estimates. Animal exposure refers to animal and animal environmental exposure documented in at least 1 case. Sources refer to the reference list in the Additional file 2: Text 1

## Results

Genetically distinct influenza A reassortants have emerged in humans on a total of 19 separate occasions since 1918. Of these, 6 strains are able to be efficiently transmitted from human-to-human, 10 are predominantly zoonotic AIVs, and 3 are predominantly zoonotic swine influenza variants.

The rate of novel strains emerging in humans has increased in recent years (Fig. 1). In the past 5 years alone, 4 novel subtypes and 3 novel variant strains have emerged in humans. A total of 14 different HA-NA combinations are known to cause human infections, with H1N1, H1N2, and H3N2 HA-NA combinations emerging multiple times since 1918 (Table 1). Zoonotic AIVs are mostly of the LPAI type (8 of 10).

Swine influenza variant viruses (H3N2v, H1N1v, H1N2v,) have all emerged in humans in the United States (US) in July 2011, December 2011 and June 2012 respectively (see Table 1), and subsequent occurrences have largely been restricted to the US and Canada. After the first zoonotic AIV human infection was reported in the US in 1979, emergence of zoonotic AIVs have been reported from the US and Canada (*n* = 3), Australia (*n* = 1) and Hong Kong (*n* = 2) and from 2013, all novel AIVs have emerged in different geographic regions in China (*n* = 3) and Taiwan (*n* = 1).

Human infections which have been associated with animal environments have been predominantly linked to a production animal species (mostly swine or avian), whilst infections caused by exposure to pets, wild animals, or laboratory animals have rarely been documented. In developed countries (North America, Europe and Australia), human cases were linked to poultry farms (112/129 cases were linked to poultry farms, 15/129 did not report details regarding exposures, 1/129 report of a laboratory exposure and 1/129 report of a pet animal exposure – see Additional file 1: Table S1 for details).

Human cases of AIV infection have typically in the past been exposed to virus via infected poultry on farms or markets, however in recent years, and particularly in China, occurrence of human infection have largely been in association with visiting live bird markets (LBMs) rather than close contact with poultry [7]. However, exposure details are missing for a large proportion of cases (see Additional file 1: Table S1 and [3, 7]). We have shown that a history of close poultry contact is far more common for human H5N1 cases than H7N9 cases [7]. In the latter case, a history of incidental poultry contact (such as walking through LBMs) is more common [7]. The reason for this difference in risk factor profile is unclear.

All zoonotic influenza infections have typically occurred infrequently, in a sporadic pattern typical of animal to human transmission without sustained human to human transmission ensuing. However there are few incidences of larger scale outbreaks which have presented with more cases than would be expected in sporadic transmission: (i) large numbers of H7N9 human cases have occurred each year since it’s emergence in 2013 (159 cases in 2013, 334 cases in 2014, 210 cases in 2015 and 99 cases in 2016 [8], (ii) the H5N1 outbreak in Egypt from 2014 to 2015 which caused 114 cases [9], and (iii) H7N7 outbreak in the Netherlands in 2003 which caused 89 cases [10]. H7N9 infections were mainly identified or reported during winter months – in 2014 and 2016 most infections (51% and 32% respectively) occurred in January, in 2015 most infections (42%) occurred in February, however in 2013 most infections (64%) occurred in April (early spring) [8].

Most zoonotic AIVs (6 of 10) and all 3 swine influenza variants typically cause mild infections in humans (see Table 1). Severe illness and fatalities are associated with only four zoonotic AIVs: high death rates have been reported for human infections with H5N6 (9/15 cases, 60%), H10N8 (2/3 cases, 67%), H7N9 (314/791 cases, 40%), and H5N1 (449/850, 53%). Mild illness is associated with infections in children – mild illness was seen for most H9N2 infections (which has a young average age of infection in humans) and for the only two cases of young children infected with H5N6 (aged 5 and 11). For H7N9 as well, young children presented with only mild infections, with more severe disease seen in older adults.

## Discussion

We have shown an increase in emergence of AIVs infecting humans in the last decade. There are several reasons which likely explain this trend: (i) improvements in zoonotic AIV case ascertainment, and (ii) a “true” increase in AIV emergence, which could be explained by an increase in AIV circulation and diversity in poultry populations, growths in the poultry industry, and increased human urbanisation.

Advances in influenza diagnostics and surveillance capabilities, as well as increased clinician awareness (particularly following the emergence and continued seasonal occurrence of H7N9), have likely contributed to better ascertainment of influenza A in humans in China. In the past, AIV reporting have likely been hindered industry and government pressure – in 1994 for example, after H9N2 was discovered in humans in China, further investigations were discouraged by Chinese government officials [11]. In contrast, China’s rapid and transparent response to the emergence of H7N9 in 2013 was widely praised by international communities. A recognised caveat however is the under ascertainment of subclinical cases: predominantly, only patients with signs and symptoms, or severe disease, present to the health system and a large proportion of cases with subclinical infection may remain unreported. A bias in the reporting of severe cases indicates that our reported CFRs are likely to be inflated. This study also finds zoonotic swine influenza viruses are more apparent in the US. However, China is the largest global producer of swine (the US is the second largest producer). Case ascertainment disparities likely explain this trend also; as there are stronger surveillance systems in the US compared to China.

This study find there have not been any reports of AIV emergence in humans in low-income, developing countries – this may also be due to case ascertainment bias. Developing countries are both unable to support high levels of active AIV surveillance (in both human and animal sectors), and highly regulated agricultural systems with the ability to enforce disease control regulations. Further, poor biosecurity measures at the human-animal interface are common in these countries, which allow for virus to more easily transmit to humans. In recent years, several countries in west and central Africa experienced a resurgence of H5N1 poultry outbreaks: Cameroon, Burkina Faso, Niger, Cote d’Ivoire, Ghana, Nigeria and Nigeria (in these countries reports of H5N1 outbreaks had last occurred in 2006 – 2009) [8]. There is a concern poultry outbreaks in these regions could escalate and cause large numbers of human infections, similar to the 2014–2015 Egypt outbreak [9]. We recommend pandemic preparedness activities focus on improving AIV control measures in developing countries.

The increase in AIV emergence in humans may also be a reflection of increased AIV persistence and diversity in poultry. Following the expansion of the poultry industry from the mid-1980s (particularly in China and some other Asian countries) a broad range of AIV lineages have evolved to circulate among domestic poultry species [12]. Prior to the emergence of H5N1 in the 1990s, all other AIV strains which caused severe outbreaks in poultry were able to be eliminated through standard control measures. This was largely due to the insufficient number of available animal hosts to sustain an outbreak. It is critical to target control measures in regions with high poultry population growth to prevent corresponding growth of AIV reservoirs in this host population.

The extent of global AIV persistence and diversity may also be reflected by recently described uncharacteristic AIV outbreaks in animals. For example in high-income countries HPAI incursions have traditionally been quickly contained and eliminated through standard disease elimination protocols, however in 2014 and 2015 HPAI caused unprecedented numbers of outbreaks in commercial poultry farms in the US, and similar outbreaks have occurred in Europe in the past year [8]. AIV transmission in domestic pets are considered to be rare, mild and unsustainable however in November 2016, LPAI H7N2 was found to cause an outbreak in 45 domestic kittens causing one fatality [13]. These events indicate a need to re-evaluate where risk of zoonotic AIV emergence is likely.

Increasing urban and agricultural encroachment into previously uninhabited areas, particularly in developing countries, may also play a role in promoting AIV transmission to humans and domestic animals from wild bird reservoir hosts. Wild migratory birds (largely from the Anseriformes and Charadriiformes families e.g. ducks, geese, gulls, waders and terns) are thought to be the traditional hosts of AIVs and require use of wetlands and lakes for breeding and wintering. Reduction of these natural habitats for wild birds and can result in birds using agricultural or urbanised areas, promoting interaction with high density farming systems (such as poultry and swine), and also humans. Such close interactions increase the risk of AIV introduction, and zoonotic disease transmission – we recommend AIV control programmes in focus on preventing such interactions particularly in African and Asian countries which are urbanizing rapidly.

Zoonotic AIV infections have been sporadically reported in developed regions such as North America, Europe and Australia, with the most recent report occurring in 2013 in Italy. All were caused by subtypes other than HPAI H5N1. Previously it was thought that only H5 and H7 HA types posed a significant pandemic risk, however strains of other HA types (H9, H6 and H10 for example) have been able to infect humans and have pandemic potential. The inability to predict which AIV types can infect humans, combined with sheer variety of AIVs circulating in poultry, makes it challenging to focus pandemic preparedness measures to specific strains. A good example is the efforts in pandemic planning globally around H5N1 from 2005 to 2009, which assumed the next pandemic would be H5 related. However, the pandemic which emerged in 2009 was a completely different virus, unrelated to H5N1. Pandemic planning should instead incorporate interventions to prevent the species jump and emergence of a human pandemic strain of influenza.

## Conclusion

We find there have been recent increases in the number of reports of AIVs infecting humans, predominantly from mainland China. We recommend pandemic preparedness measures focus on preventing zoonotic disease emergence, specifically the strengthening of control efforts to reduce (i) potential introductions of AIVs into poultry populations, (ii) subsequent spread within the poultry sector, and (iii) virus transmission at the human-animal interface (particularly within LBMs). Regional disparities should also be considered. Low-income countries which are undergoing rapid commercialisation of their poultry sector are at highest risk of harbouring AIVs, and are unlikely to detect nor adequately control AIV spread.

## Additional files

Additional file 1: Table S1. Epidemiological features of reported outbreaks or isolate cases of distinct avian influenza serotypes. This is a table showing the information we have collected on reported outbreaks or isolate cases of distinct avian influenza strains. (DOCX 70 kb)

Additional file 2: Text 1. The following is the reference list for Table 1 and Additional file 1: Table S1. This is a list of references showing where information was collected from – the number of the reference corresponds to numbers in Table 1 and Additional file 1: Table S1. (DOCX 22 kb)

## Abbreviations

AIV: Avian influenza virus
CDC: Centres for disease control and prevention
CFR: Case fatality rate
EID: Emerging infectious disease
HA: Hemagglutinin
HPAI: Highly pathogenic avian influenza
LBM: Live bird market
LPAI: Low pathogenic avian influenza
US: United States
WHO: World Health Organisations
