# Supplementary material for: An overview of the epidemiology and emergence of influenza A infection in humans over time
Source: Arch Public Health. 2017 Mar 27;75:15. doi: 10.1186/s13690-017-0182-z (PMC5366997; doi:10.1186/s13690-017-0182-z)
Supplement: Supplementary file 1 — Epidemiological features of reported outbreaks or isolate cases of distinct avian influenza serotypes. This is a table showing the information we have collected on reported outbreaks or isolate cases of distinct avian influenza strains. (DOCX 70 kb) [file 13690_2017_182_MOESM1_ESM.docx]

**Supplementary Table 1.** Epidemiological features of reported outbreaks or isolate cases of distinct avian influenza strains

| Year of incidence | Country of incidence | Person/s affected | Clinical signs | Exposure history | Subtype | Sources |
| --- | --- | --- | --- | --- | --- | --- |
| 1979 | United States | Laboratory worker | Conjunctivitis | Patient was sneezed on by a seal experimentally infected with H7N7 | H7N7 | [35] |
| 1996 | England | Adult female | Conjunctivitis | Patient owned pet ducks of various breeds, which have mixed with visiting wild birds (e.g. mallards and Canada geese) | LPAI H7N7 | [53] |
| 1998 – 1999 | China | 5 cases: 4-year-old male, 1-year-old female, 36-year-old female, 1-year-old male, 75-year-old male | Acute respiratory disease. All had full recoveries | Unknown. Cases identified from seroprevalence surveillance | LPAI H9N2 | [39, 54] |
| 1999 | Hong Kong | Three children: 4-year-old female, 1-year-old female, 2-year-old male | Fever, cough, vomiting. All have fully recovered | Unknown poultry contact for first two cases | LPAI H9N2 | [39, 54, 55] |
| 2002 | United States | Poultry workers | Unknown – diagnosed on serology | LPAI H7N2 outbreak in Virginia in 2002 which resulted in the culling of 4.7 million turkeys and chickens. Patient was involved in culling. | LPAI H7N2 | [30] |
| 2002-2003 | Italy | 7 poultry workers | Unknown – cases were identified by large scale serological surveillance study of poultry workers | Anti-H7 antibodies found in 3.8% of serum samples collected from poultry workers during 2003 when LPAI H7N3 virus was circulating. All seropositive subjects had close direct physical contact with turkeys or chickens | LPAI H7N3 | [56] |
| 2003 | Hong Kong | 5-year-old male | Fever, cough, recovered without complication | No direct contact with poultry | LPAI H9N2 | [55] |
| 2003 | United States | Adult male | Upper and lower respiratory tract illness | No epidemiological link to any birds or bird environments | LPAI H7N2 | [57] |
| 2003 | Netherlands | 89 humans. 5 veterinarians, 54 poultry cullers, 13 poultry farmers, 3 family members of infected poultry farmers, 14 others | Most cases had conjunctivitis (89), 6 cases had ILI symptoms, 2 cases had red-eye. 1 case had fatal acute respiratory distress syndrome. | All cases had close contact with poultry except for 3 family members of infected poultry workers. | HPAI H7N7 | [37, 58] |
| 2004 | Canada | Two poultry workers | Conjunctivitis and mild influenza-like illness | Both had been in contact with HPAI H7N3 infected poultry | HPAI H7N3 | [27] |
| 2004 | Egypt | 2 children | Mild symptoms | Father of one of the children was linked to a LBM which was later tested positive to the virus | H10N7 | [59] |
| 2006 | United Kingdom | Poultry worker | Conjunctivitis | Outbreak of LPAI H7N3 in poultry flock in England. No exposure details were reported for this UK poultry worker | LPAI H7N3 | [60] |
| 2007 | United Kingdom | 14 humans | Mild respiratory symptoms | All 14 individuals had been exposed to LPAI H7N2 infected birds (no details provided) | LPAI H7N2 | [31] |
| 2008 | China | 3-month-old girl (leukaemia patient) | Cough, nasal obstruction, and  vomiting | Mother had history of contact with live poultry. | LPAI H9N2 | [54] |
| 2009 | China | 47-year-old woman (immune-compromised patient) | Fever, chills, cough, and  dyspnoea | Visited LBM 4 days before symptom onset. Could not recall contact with poultry | LPAI H9N2 | [54] |
| 2010 | Australia | 7 abattoir workers | Conjunctivitis and minor upper respiratory tract symptoms | Workers were involved in processing chicken from a commercial chicken farm which had earlier tested positive to LPAI H10N7 | LPAI H10N7 | [22] |
| 2011 | Bangladesh | 4-year-old female | Respiratory signs and fever | Patient participated in preparing raw chicken for cooking | LPAI H9N2 | [61] |
| 2012 | Mexico | Two poultry workers | Conjunctivitis | Both poultry workers worked in a HPAI H7N3 infected poultry | HPAI H7N3 | [28] |
| 2012 | Taiwan | 2 poultry vendors, 4 poultry farmers, 1 non-poultry worker | Subclinical | H7N3 antibodies found in 7 individuals in serological surveillance in Taiwan following 2011 LPAI H7N3 outbreaks in poultry | LPAI H7N3 | [7] |
| 2013 | Taiwan | 20-year-old female | Respiratory signs | The patient reported no recent handling of raw poultry meat and no contact with any poultry in the past year. Fecal samples collected from a nearby flock of 60 poultry tested negative for influenza virus A | LPAI H6N1 | [6] |
| 2013 | China | 86-year-old male | Respiratory signs and fever | Patient had not come into direct contact with live poultry | LPAI H9N2 | [62] |
| 2013 | China | 7-year-old male | Respiratory tract signs and fever. Also vomiting and diarrhoea | No history of direct contact with birds | LPAI H9N2 | [63] |
| 2013 | Italy | 3 poultry workers | Conjunctivitis | Poultry workers involved in breeding, cleaning and culling of HPAI H7N7 infected poultry | HPAI H7N7 | [36] |
| 2013-2014 | China | 3 humans | Severe disease, 2 fatalities | The first patient had visited a LBM 4 days before illness onset but had no direct contact with poultry [64]. The exposure history is not clear for the second case. The third case had also visited a LBM before illness onset. | LPAI H10N8 | Promed Archive numbers: 20140214.2277024; 20140126.2233164; 20131217.2121718 |
| 2014 | China | 5-year-old female | Influenza-like illness (fever and sore throat). Recovered in 2 days | Walked past areas (restaurant, market) where live poultry were held in cages 2-6 days before illness onset. | HPAI H5N6 | [1, 65] |
| 2014 | China | 49-year-old male | Severe respiratory symptoms,Fatal | This patient was a poultry dealer and housed unsold poultry in his backyard. During April 13-17, birds in his home were found to have died, and reports indicate detection of H5N6 virus | HPAI H5N6 | [66] |
| 2014 | China | 59-year-old male | Severe respiratory symptoms,discharged | Regularly purchased and handled live poultry in LBMs during the weeks before illness onset | HPAI H5N6 | [67] |
| 2015 | Egypt | 3-year-old male | No details | History of contact with apparently healthy backyard poultry. H9N2 positive poultry have been detected in Egypt since 2011 | LPAI H9N2 | Promed Archive Number: 20150215.3168314 |
| 2015 | Bangladesh | 4-year-old female | Mild illness | The case had close contact with poultry, including sick quail, prior to her onset of illness | LPAI H9N2 | Promed Archive Number: 20150923.3666519 |
| 2015 | Egypt | 1-year-old female | Mild illness | No information available | LPAI H9N2 | [WHO, Influenza at the human-animal interface – 23 June 2015](http://www.who.int/influenza/human_animal_interface/Influenza_Summary_IRA_HA_interface_23_June_2015.pdf) |
| 2015 | China | 4-year-old female | Mild illness | Exposure to Live animal market | LPAI H9N2 | [WHO, Influenza at the human-animal interface – 14 December 2015](http://www.who.int/influenza/human_animal_interface/Influenza_Summary_IRA_HA_interface_14_Dec_2015.pdf) |
| 2015 | Egypt | 7-year-old male | Mild illness | History of exposure to live bird market poultry | LPAI H9N2 | Promed Archive Number: 20150522.3378923 |
| 2015 | China | 2-year-old male | Mild illness | No information available | LPAI H9N2 | [WHO, Influenza at the human-animal interface – 14 December 2015](http://www.who.int/influenza/human_animal_interface/Influenza_Summary_IRA_HA_interface_14_Dec_2015.pdf) |
| 2015 | China | 15-year-old female | Mild illness | No known exposure | LPAI H9N2 | [WHO, Influenza at the human-animal interface – 14 December 2015](http://www.who.int/influenza/human_animal_interface/Influenza_Summary_IRA_HA_interface_14_Dec_2015.pdf) |
| 2015 | China | 1-year-old female | Mild illness | No information available | LPAI H9N2 | [WHO, Influenza at the human-animal interface – 14 December 2015](http://www.who.int/influenza/human_animal_interface/Influenza_Summary_IRA_HA_interface_14_Dec_2015.pdf) |
| 2015 | Bangladesh | 48-year-old male | Fever, runny nose, headache and myalgia. | Poultry worker in a market. One day prior to illness onset, the case reported handling sick poultry | LPAI H9N2 | [WHO, Influenza at the human-animal interface – 20 January 2016](http://www.who.int/influenza/human_animal_interface/Influenza_Summary_IRA_HA_interface_20_Jan_2016.pdf) |
| 2015 | China | 43-year-old male | Severe respiratory symptoms. Fatal | Visited a food market that sold live poultry  three days before onset of illness but had not been exposed to poultry at this time. Hunted two wild birds from the Napahai wetland the subsequent day. Samples from nearby LBMs tested positive for H5N6. | HPAI H5N6 | [68, 69] [WHO, Disease outbreak news – 12 February 2015](http://www.who.int/csr/don/12-february-2015-avian-influenza/en/) |
| 2015 | China | 37-year-old female | Severe respiratory symptoms. Fatal | No information available | HPAI H5N6 | [68] [WHO, Disease outbreak news – 14 July 2015](http://www.who.int/csr/don/14-july-2015-avian-influenza/en/) |
| 2015 | China | 25-year-old male | Hospitalized in critical condition. Fatal | History of visiting LBM | HPAI H5N6 | [70] [WHO, Disease outbreak news – 11 January 2016](http://www.who.int/csr/don/11-january-2016-avian-influenza-china/en/) |
| 2015 | China | 42-year-old male | Hospitalized. Fatal | History of visiting LBM | HPAI H5N6 | Promed Archive Number: 20160127.3971269, [WHO, Disease outbreak news – 11 January 2016](http://www.who.int/csr/don/11-january-2016-avian-influenza-china/en/) |
| 2015 | China | 26-year-old female | Critical condition. Fatal | Handled duck meat | HPAI H5N6 | [WHO, Influenza at the human-animal interface – 20 January 2016](http://www.who.int/influenza/human_animal_interface/Influenza_Summary_IRA_HA_interface_20_Jan_2016.pdf) |
| 2015 | China | 40-year-old female | Hospitalized in critical condition. Fatal | Exposure to live poultry. | HPAI H5N6 | [WHO, Influenza at the human-animal interface – 20 January 2016](http://www.who.int/influenza/human_animal_interface/Influenza_Summary_IRA_HA_interface_20_Jan_2016.pdf) |
| 2016 | China | 31-year-old female | n/a | No information available | HPAI H5N6 | [WHO, Influenza at the human-animal interface – 20 January 2016](http://www.who.int/influenza/human_animal_interface/Influenza_Summary_IRA_HA_interface_20_Jan_2016.pdf) |
| 2016 | China | 40-year-old female | Hospitalized in critical condition. | History of visiting LBM | HPAI H5N6 | Promed Archive Number: 20160315.4095095 |
| 2016 | China | 35-year-old male | Hospitalized. | No information available | HPAI H5N6 | Promed Archive Number: 20160423.4178977 |
| 2016 | China | 11-year-old female | Hospitalized. Stable condition. | History of poultry contact before onset | HPAI H5N6 | Promed Archive Number: 20160428.4186019 |
| 2016 | China | 50-year-old male | Critical condition. | No information available | HPAI H5N6 | Promed Archive Number: 20160610.4275291, [WHO, Disease outbreak news – 8 June 2016](http://www.who.int/csr/don/08-june-2016-ah5n6-china/en/) |
| 2016 | China | 65-year-old female | Hospitalized. Fatal | Exposure to live poultry before symptom onset | HPAI H5N6 | [WHO, Disease outbreak news – 10 May 2016](http://www.who.int/csr/don/10-may-2016-ah5n6-china/en/) |
| 2016 | China | 57-year-old female | Recurrent productive cough with fatigue and shortness of breath (chronic underlying conditions) | No information available | LPAI H9N2 | Promed Archive Number: 20160318.4101269 |
| 2016 | Egypt | 18-year-old male | Influenza-like illness | Exposure to live poultry at market 2 weeks prior to onset of illness | LPAI H9N2 | [WHO, Influenza at the human-animal interface – 9 May 2016](http://who.int/influenza/human_animal_interface/Influenza_Summary_IRA_HA_interface_05_09_2016.pdf) |
| 2016 | China | 4-year-old female | Hospitalized. Serious condition. | No information available | LPAI H9N2 | Promed Archive Number: 20160621.4299567 |
| 2016 | China | 29-year-old female | Not described | No information available | LPAI H9N2 | [Flutrackers](https://flutrackers.com/forum/forum/china-h5n1-h5n8-h5n6-h5n3-h5n2-h10n8-outbreak-tracking/759374-china-guangdong-province-announces-1-new-h9n2-bird-flu-case-from-zhongshan-city-august-25-2016) |
| 2016 | China | 10-month-old | Not described | No information available | LPAI H9N2 | Promed Archive Number: 20160901.4451359 |
| 2016 | China | 4-year-old female | Not described | No information available | LPAI H9N2 | Promed Archive Number: 20160912.4481431 |
