# Supplementary material for: An overview of the epidemiology and emergence of influenza A infection in humans over time
Source: Arch Public Health. 2017 Mar 27;75:15. doi: 10.1186/s13690-017-0182-z (PMC5366997; doi:10.1186/s13690-017-0182-z)
Supplement: Supplementary file 2 — The following is the reference list for Table 1 and Additional file 1: Table S1. This is a list of references showing where information was collected from – the number of the reference corresponds to numbers in Table 1 and Additional file 1: Table S1. (DOCX 22 kb) [file 13690_2017_182_MOESM2_ESM.docx]

**Supplementary text 1.** The following is the reference list for **Table 1** and **Supplementary Table 1**.

1. Chen T, Zhang R: Symptoms seem to be mild in children infected with avian influenza A (H5N6) and other subtypes. Journal of Infection 2015, 71(6):702-703.

2. PromED-mail. Program for Monitoring Emerging Diseases. <http://promedmail.org/>. Accessed April 2016.

3. FAO FaAO. Epidemiology of Avian Influenza. <http://www.fao.org/avianflu/en/clinical.html>. Accessed 17 July 2014.

4. Benson DA, Karsch-Mizrachi I, Lipman DJ, Ostell J, Sayers EW: GenBank. Nucleic Acids Res 2009, 37(Database issue):D26-31.

5. Yu Z, Gao X, Wang T, Li Y, Li Y, Xu Y, Chu D, Sun H, Wu C, Li S *et al*: Fatal H5N6 avian influenza virus infection in a domestic cat and wild birds in china. Scientific Reports 2015, 5.

6. Wei SH, Yang JR, Wu HS, Chang MC, Lin JS, Lin CY, Liu YL, Lo YC, Yang CH, Chuang JH *et al*: Human infection with avian influenza A H6N1 virus: an epidemiological analysis. Lancet Respir Med 2013, 1(10):771-778.

7. Huang SY, Yang JR, Lin YJ, Yang CH, Cheng MC, Liu MT, Wu HS, Chang FY: Serological comparison of antibodies to avian influenza viruses, subtypes H5N2, H6N1, H7N3 and H7N9 between poultry workers and non-poultry workers in Taiwan in 2012. Epidemiol Infect 2015.

8. Cheung CL, Vijaykrishna D, Smith GJ, Fan XH, Zhang JX, Bahl J, Duan L, Huang K, Tai H, Wang J *et al*: Establishment of influenza A virus (H6N1) in minor poultry species in southern China. J Virol 2007, 81(19):10402-10412.

9. Bui C, Bethmont A, Chughtai AA, Gardner L, Sarkar S, Hassan S, Seale H, MacIntyre CR: A Systematic Review of the Comparative Epidemiology of Avian and Human Influenza A H5N1 and H7N9 - Lessons and Unanswered Questions. Transbound Emerg Dis 2016, 63(6):602-620.

10. Food and Agricultural Organisation of the United Nations (FAO). H7N9 situation update - 20 July 2016. <http://www.fao.org/ag/againfo/programmes/en/empres/h7n9/situation_update.html>. Accessed Sept 14 2016.

11. Bethmont A, Bui CM, Gardner L, Sarkar S, Chughtai AA, Macintyre CR: Quantified degree of poultry exposure differs for human cases of avian influenza H5N1 and H7N9. Epidemiol Infect 2016, 144(12):2633-2640.

12. Farooqui A, Liu W, Zeng T, Liu Y, Zhang L, Khan A, Wu X, Wu R, Wu S, Huang L *et al*: Probable Hospital Cluster of H7N9 Influenza Infection. New England Journal of Medicine 2016, 374(6):596-598.

13. Liu Y, Paquette SG, Zhang L, Leon AJ, Liu W, Xiuming W, Huang L, Wu S, Lin P, Chen W *et al*: The Third Wave: H7N9 Endemic Reassortant Viruses and Patient Clusters. J Infect Dev Ctries 2015, 9(2):122-127.

14. Chen H, Liu S, Liu J, Chai C, Mao H, Yu Z, Tang Y, Zhu G, Chen HX, Zhu C *et al*: Nosocomial Co-Transmission of Avian Influenza A(H7N9) and A(H1N1)pdm09 Viruses between 2 Patients with Hematologic Disorders. Emerging Infectious Diseases 2016, 22(4).

15. Su S, Zhou P, Fu X, Wang L, Hong M, Lu G, Sun L, Qi W, Ning Z, Jia K *et al*: Virological and epidemiological evidence of avian influenza virus infections among feral dogs in live poultry markets, china: a threat to human health? Clin Infect Dis 2014, 58(11):1644-1646.

16. Komadina N, McVernon J, Hall R, Leder K: A historical perspective of influenza A(H1N2) virus. Emerg Infect Dis 2014, 20(1):6-12.

17. Centers for Disease Control and Prevention (CDC). Reported Infections with Variant Influenza Viruses in the United States since 2005. <http://www.cdc.gov/flu/swineflu/variant-cases-us.htm>. Accessed September 2016.

18. Vincent A, Awada L, Brown I, Chen H, Claes F, Dauphin G, Donis R, Culhane M, Hamilton K, Lewis N *et al*: Review of influenza A virus in swine worldwide: a call for increased surveillance and research. Zoonoses Public Health 2014, 61(1):4-17.

19. Centers for Disease Control and Prevention (CDC). Case Count: Detected U.S. Human Infections with H3N2v by State since August 2011. <http://www.cdc.gov/flu/swineflu/h3n2v-case-count.htm>. Accessed July 16 2016.

20. Jhung MA, Epperson S, Biggerstaff M, Allen D, Balish A, Barnes N, Beaudoin A, Berman L, Bidol S, Blanton L *et al*: Outbreak of variant influenza A(H3N2) virus in the United States. Clinical Infectious Diseases 2013, 57(12):1703-1712.

21. Dawood FS, Jain S, Finelli L, Shaw MW, Lindstrom S, Garten RJ, Gubareva LV, Xu X, Bridges CB, Uyeki TM: Emergence of a novel swine-origin influenza A (H1N1) virus in humans. N Engl J Med 2009, 360(25):2605-2615.

22. Arzey GG, Kirkland PD, Arzey KE, Frost M, Maywood P, Conaty S, Hurt AC, Deng YM, Iannello P, Barr I *et al*: Influenza virus a (H10N7) in chickens and poultry abattoir workers, Australia. Emerging Infectious Diseases 2012, 18(5):814-816.

23. Bodewes R, Bestebroer TM, Van Der Vries E, Verhagen JH, Herfst S, Koopmans MP, Fouchier RA, Pfankuche VM, Wohlsein P, Siebert U *et al*: Avian influenza a(H10n7) virus–associated mass deaths among harbor seals. Emerging Infectious Diseases 2015, 21(4):720-722.

24. Hall JS, Bentler KT, Landolt G, Elmore SA, Minnis RB, Campbell TA, Barras SC, Root JJ, Pilon J, Pabilonia K *et al*: Influenza infection in wild raccoons. Emerging Infectious Diseases 2008, 14(12):1842-1848.

25. Van Kerkhove MD, Hirve S, Koukounari A, Mounts AW: Estimating age-specific cumulative incidence for the 2009 influenza pandemic: a meta-analysis of A(H1N1)pdm09 serological studies from 19 countries. Influenza Other Respir Viruses 2013, 7(5):872-886.

26. Goldstein T, Mena I, Anthony SJ, Medina R, Robinson PW, Greig DJ, Costa DP, Lipkin WI, Garcia-Sastre A, Boyce WM: Pandemic H1N1 influenza isolated from free-ranging Northern Elephant Seals in 2010 off the central California coast. PLoS One 2013, 8(5):e62259.

27. Tweed SA, Skowronski DM, David ST, Larder A, Petric M, Lees W, Li Y, Katz J, Krajden M, Tellier R *et al*: Human illness from avian influenza H7N3, British Columbia. Emerg Infect Dis 2004, 10(12):2196-2199.

28. Lopez-Martinez I, Balish A, Barrera-Badillo G, Jones J, Nunez-Garcia TE, Jang Y, Aparicio-Antonio R, Azziz-Baumgartner E, Belser JA, Ramirez-Gonzalez JE *et al*: Highly pathogenic avian influenza A(H7N3) virus in poultry workers, Mexico, 2012. Emerg Infect Dis 2013, 19(9):1531-1534.

29. Food and Agriculture Organisation of the United Nations. EMPRES-i Global Animal Disease Information System. <http://www.fao.org/EMPRES/default.htm>. Accessed 20 October 2016.

30. Edwards LE, Terebuh P, Adija A: Serological diagnosis of human infection with avian influenza A (H7N2) virus [Abstract 60, Session 44]. In: *International Conference on Emerging Infectious Diseases 2004: 2004; Atlanta, Georgia, February 22--March 3*; 2004.

31. Eames KT, Webb C, Thomas K, Smith J, Salmon R, Temple JM: Assessing the role of contact tracing in a suspected H7N2 influenza A outbreak in humans in Wales. BMC Infect Dis 2010, 10:141.

32. Kwon TY, Lee SS, Kim CY, Shin JY, Sunwoo SY, Lyoo YS: Genetic characterization of H7N2 influenza virus isolated from pigs. Vet Microbiol 2011, 153(3-4):393-397.

33. Goddard NL, Joseph CA, Watson JM, Zambon M: Epidemiological features of a new strain of the influenza A virus--influenza A (H1N2) circulating in England and its public health implications. Virus Res 2004, 103(1-2):53-54.

34. Xu X, Smith CB, Mungall BA, Lindstrom SE, Hall HE, Subbarao K, Cox NJ, Klimov A: Intercontinental circulation of human influenza A(H1N2) reassortant viruses during the 2001-2002 influenza season. J Infect Dis 2002, 186(10):1490-1493.

35. Webster RG, Geraci J, Petursson G, Skirnisson K: Conjunctivitis in human beings caused by influenza A virus of seals. N Engl J Med 1981, 304(15):911.

36. Puzelli S, Rossini G, Facchini M, Vaccari G, Di Trani L, Di Martino A, Gaibani P, Vocale C, Cattoli G, Bennett M *et al*: Human infection with highly pathogenic A(H7N7) avian influenza virus, Italy, 2013. Emerg Infect Dis 2014, 20(10):1745-1749.

37. Koopmans M, Wilbrink B, Conyn M, Natrop G, van der Nat H, Vennema H, Meijer A, van Steenbergen J, Fouchier R, Osterhaus A *et al*: Transmission of H7N7 avian influenza A virus to human beings during a large outbreak in commercial poultry farms in the Netherlands. Lancet 2004, 363(9409):587-593.

38. Murcia PR, Wood JL, Holmes EC: Genome-scale evolution and phylodynamics of equine H3N8 influenza A virus. J Virol 2011, 85(11):5312-5322.

39. Guo Y, Li J, Cheng X: [Discovery of men infected by avian influenza A (H9N2) virus]. Zhonghua Shi Yan He Lin Chuang Bing Du Xue Za Zhi 1999, 13(2):105-108.

40. Al-Garib S, Agha A, Al-Mesilaty L: Low pathogenic avian influenza H9N2: World-wide distribution. World's Poultry Science Journal 2016, 72(1):125-136.

41. Yu H, Hua RH, Wei TC, Zhou YJ, Tian ZJ, Li GX, Liu TQ, Tong GZ: Isolation and genetic characterization of avian origin H9N2 influenza viruses from pigs in China. Vet Microbiol 2008, 131(1-2):82-92.

42. Lai S, Qin Y, Cowling BJ, Ren X, Wardrop NA, Gilbert M, Tsang TK, Wu P, Feng L, Jiang H *et al*: Global epidemiology of avian influenza A H5N1 virus infection in humans, 1997-2015: A systematic review of individual case data. The Lancet Infectious Diseases 2016.

43. Guo YJ, Xu XY, Cox NJ: Human influenza A (H1N2) viruses isolated from China. J Gen Virol 1992, 73 ( Pt 2):383-387.

44. Kung H, Jen K, Yuan W, Tien S, Chu C: Influenza in China in 1977: recurrence of influenzavirus A subtype H1N1. Bull World Health Organ 1978, 56(6):913.

45. World Health Organization (WHO). Influenza (Seasonal) Fact sheet No211 (March 2014). <http://www.who.int/mediacentre/factsheets/fs211/en/>. Accessed September 2016.

46. Finkelman BS, Viboud C, Koelle K, Ferrari MJ, Bharti N, Grenfell BT: Global patterns in seasonal activity of influenza A/H3N2, A/H1N1, and B from 1997 to 2005: viral coexistence and latitudinal gradients. PLoS One 2007, 2(12):e1296.

47. Brockwell-Staats C, Webster RG, Webby RJ: Diversity of influenza viruses in swine and the emergence of a novel human pandemic influenza A (H1N1). Influenza Other Respir Viruses 2009, 3(5):207-213.

48. Chen Y, Mo YN, Zhou HB, Wei ZZ, Wang GJ, Yu QX, Xiao X, Yang WJ, Huang WJ: Emergence of human-like H3N2 influenza viruses in pet dogs in Guangxi, China. Virol J 2015, 12:10.

49. Lu Y, Roberts B, Essex M: Emerging infections in Asia. New York, N.Y.: New York, N.Y. : Springer; 2008.

50. Viboud C, Simonsen L, Fuentes R, Flores J, Miller MA, Chowell G: Global Mortality Impact of the 1957-1959 Influenza Pandemic. J Infect Dis 2016, 213(5):738-745.

51. Taubenberger JK, Morens DM: Pandemic influenza--including a risk assessment of H5N1. Rev Sci Tech 2009, 28(1):187-202.

52. Taubenberger JK, Morens DM: 1918 Influenza: the mother of all pandemics. Emerg Infect Dis 2006, 12(1):15-22.

53. Kurtz J, Manvell RJ, Banks J: Avian influenza virus isolated from a woman with conjunctivitis. Lancet 1996, 348(9031):901-902.

54. Cheng VC, Chan JF, Wen X, Wu WL, Que TL, Chen H, Chan KH, Yuen KY: Infection of immunocompromised patients by avian H9N2 influenza A virus. J Infect 2011, 62(5):394-399.

55. Butt KM, Smith GJ, Chen H, Zhang LJ, Leung YH, Xu KM, Lim W, Webster RG, Yuen KY, Peiris JS *et al*: Human infection with an avian H9N2 influenza A virus in Hong Kong in 2003. J Clin Microbiol 2005, 43(11):5760-5767.

56. Puzelli S, Di Trani L, Fabiani C, Campitelli L, De Marco MA, Capua I, Aguilera JF, Zambon M, Donatelli I: Serological analysis of serum samples from humans exposed to avian H7 influenza viruses in Italy between 1999 and 2003. J Infect Dis 2005, 192(8):1318-1322.

57. Ostrowsky B, Huang A, Terry W, Anton D, Brunagel B, Traynor L, Abid S, Johnson G, Kacica M, Katz J *et al*: Low pathogenic avian influenza A (H7N2) virus infection in immunocompromised adult, New York, USA, 2003. Emerg Infect Dis 2012, 18(7):1128-1131.

58. Fouchier RA, Schneeberger PM, Rozendaal FW, Broekman JM, Kemink SA, Munster V, Kuiken T, Rimmelzwaan GF, Schutten M, Van Doornum GJ *et al*: Avian influenza A virus (H7N7) associated with human conjunctivitis and a fatal case of acute respiratory distress syndrome. Proc Natl Acad Sci U S A 2004, 101(5):1356-1361.

59. Pan American Health Organization: Avian influenza virus A (H10N7) circulating among humans in Egypt. In: *EID Weekly Updates, Emerging and Reemerging Infectious Diseases, Region of the Americas* vol. Vol. 2, No. 18—7 May 2004; 2004.

60. Nguyen-Van-Tam JS, Nair P, Acheson P, Baker A, Barker M, Bracebridge S, Croft J, Ellis J, Gelletlie R, Gent N *et al*: Outbreak of low pathogenicity H7N3 avian influenza in UK, including associated case of human conjunctivitis. Euro Surveill 2006, 11(5):E060504 060502.

61. Shanmuganatham K, Feeroz MM, Jones-Engel L, Smith GJ, Fourment M, Walker D, McClenaghan L, Alam SM, Hasan MK, Seiler P *et al*: Antigenic and molecular characterization of avian influenza A(H9N2) viruses, Bangladesh. Emerg Infect Dis 2013, 19(9).

62. South China Morning Post. Hong Kong sees first case of H9N2 avian flu in four years. <http://www.scmp.com/news/hong-kong/article/1393266/86-year-old-man-infected-h9n2-avian-flu>. Accessed 27 August 2015.

63. Huang Y, Li X, Zhang H, Chen B, Jiang Y, Yang L, Zhu W, Hu S, Zhou S, Tang Y *et al*: Human infection with an avian influenza A (H9N2) virus in the middle region of China. J Med Virol 2015.

64. Chen H, Yuan H, Gao R, Zhang J, Wang D, Xiong Y, Fan G, Yang F, Li X, Zhou J *et al*: Clinical and epidemiological characteristics of a fatal case of avian influenza A H10N8 virus infection: A descriptive study. The Lancet 2014, 383(9918):714-721.

65. Zhang R, Chen T, Ou X, Liu R, Yang Y, Ye W, Chen J, Yao D, Sun B, Zhang X *et al*: Clinical, epidemiological and virological characteristics of the first detected human case of avian influenza A(H5N6) virus. Infection, Genetics and Evolution 2016, 40:236-242.

66. Pan M, Gao R, Lv Q, Huang S, Zhou Z, Yang L, Li X, Zhao X, Zou X, Tong W *et al*: Human infection with a novel, highly pathogenic avian influenza A (H5N6) virus: Virological and clinical findings. Journal of Infection 2016, 72(1):52-59.

67. Yang ZF, Mok CKP, Peiris JSM, Zhong NS: Human infection with a novel avian influenza A(H5N6) virus. New England Journal of Medicine 2015, 373(5):487-489.

68. Xu W, Li H, Jiang L: Human infection with a highly pathogenic avian influenza A (H5N6) virus in Yunnan province, China. Infect Dis (Lond) 2016, 48(6):477-482.

69. Xu W, Li X, Bai T, Zhao X, Zhao X, Zhang Y, Guo J, Li Z, Yang L, Wang D *et al*: A fatal case of infection with a further reassortant, highly pathogenic avian influenza (HPAI) H5N6 virus in Yunnan, China. Infection, Genetics and Evolution 2016, 40:63-66.

70. Zhang YL, Yang SG, Li G, Yuan J, Ding H, Mao C, Liu Q: Clinical and epidemiological characteristics of a case of avian influenza A H5N6 virus infection. Journal of Infection 2016.
